# Methodologies for the Determination of Blood Alpha1 Antitrypsin Levels: A Systematic Review

**DOI:** 10.3390/jcm10215132

**Published:** 2021-10-31

**Authors:** Borja Ruiz-Duque, Lucía Bañuls, Rocio Reinoso-Arija, Laura Carrasco-Hernandez, Candelaria Caballero-Eraso, Francisco Dasí, José Luis Lopez-Campos

**Affiliations:** 1 Unidad Médico-Quirúrgica de Enfermedades Respiratorias, Instituto de Biomedicina de Sevilla (IBiS), Hospital Universitario Virgen del Rocío, Universidad de Sevilla, 41013 Seville, Spain; borja_994@hotmail.com (B.R.-D.); rocioreari@gmail.com (R.R.-A.); lauracarrascohdez@gmail.com (L.C.-H.); ccaballero-ibis@us.es (C.C.-E.); 2 Departamento de Fisiología, Instituto de Investigación Sanitaria INCLIVA, Facultad de Medicina, Universidad de Valencia, 46010 Valencia, Spain; lucia.banyuls.soto@gmail.com (L.B.); Francisco.Dasi@uv.es (F.D.); 3 Centro de Investigación Biomédica en Red de Enfermedades Respiratorias (CIBERES), Instituto de Salud Carlos III, 28029 Madrid, Spain

**Keywords:** alpha1-antitripsin, serum, plasma, blood concentration, nephelometry, turbidimetry

## Abstract

Background: The study of hematic concentrations of alpha1 antitrypsin (AAT) is currently one step in the diagnosis of AAT deficiency. To try to clarify the relevance of the laboratory techniques, we carried out a systematic review of the literature. Methods: Studies evaluating the quantification of AAT in peripheral blood were searched in PubMed in July 2021. The selection criteria included (1) any type of study design that included a quantification of AAT in peripheral blood; (2) studies written in English or Spanish; (3) studies evaluating human beings; and (4) studies involving adults. Results: Out of 207 studies, the most frequently used techniques were nephelometry (43.9%), followed by ELISA (19.8%) and turbidimetry (13.5%). Altogether, 182 (87.9%) cases expressed their results in units of gram, while 16 (7.7%) articles expressed them in units of mole. Only 2.9% articles referred to the standard used, 43.5% articles indicated the commercial kit used, and 36.2% indicated the analyzer used. Conclusions: The technical aspects of these determinations are not always reported in the literature. Journals should be attentive to these technical requirements and ensure that they are included in the works in which AAT is determined in order to ensure a correct interpretation of the study findings.

## 1. Introduction

Currently, the early identification and the correct diagnosis of alpha1 antitrypsin (AAT) deficiency (AATD) constitutes one of the great challenges in the management of this clinical condition. The figures for underdiagnosis in the community reach high levels and the number of potential cases not identified is striking [1,2]. The consequences of this insufficient diagnosis have a direct impact on the care of patients who do not receive adequate evaluation and treatment. Furthermore, the lack of identification of patients with AATD has as a consequence the non-performance of family screening and, therefore, the non-identification of patients at an early age [3]. Consequently, it is not possible to establish preventive measures in sick subjects to avoid the appearance and progression of associated lesions. Finally, it has recently been described the considerable burden of this underdiagnosed condition [4,5].

At present, there are numerous algorithms for the identification of AATD. Although these algorithms have some variability, most of them perform a determination of AAT in peripheral blood at some point during the diagnostic process [6,7]. Interestingly, the determination of AAT in peripheral blood has some technical conditions that are relevant when interpreting the results well. However, many investigators do not take these differences into account and it is generally assumed that the determination of AAT in blood is equally valid regardless of the laboratory method used [8,9,10].

To try to clarify the relevance of the laboratory techniques in the determination of AAT, the first step would be to identify which laboratory techniques are used in the study of hematic concentrations of AAT. With this objective, we carried out a systematic review of the literature to help us identify the techniques used, the types of samples, the units of measurement in which the results are expressed, and other technical conditioning factors that may influence the determination of AAT in peripheral blood. This review allowed us to review these techniques and their technical considerations that will help the clinician and investigator to better interpret the results of these determinations.

## 2. Methods

The present analysis comprises a systematic review of studies evaluating the quantification of AAT in peripheral blood. Due to the non-interventional nature of the procedures evaluated, a registration number was not considered to be necessary. On July 2021, we completed a systematic search in PubMed including articles since the year 2000, using the following terms “antitrypsin [title] AND (serum [title/abstract] OR plasma [title/abstract]) NOT (review [PT] OR case reports [PT] OR editorial [PT]) NOT (pediatr* OR paediatr* OR infants)”. All identified abstracts were retrieved and evaluated. The selection criteria included: (1) any type of study design that included a quantification of AAT in peripheral blood; (2) being written in English or Spanish; (3) evaluating human beings; and (4) involving adults. Accordingly, preclinical or animal model studies and pediatric studies were not considered. Additionally, we excluded the following trials: (1) studies available only in a congress abstract form; (2) studies which were not original clinical research (i.e., systematic or narrative reviews).

The selected studies were downloaded in full and information on the determination of blood AAT was registered in a Microsoft Excel sheet (Microsoft Corporation, Redmond, WA, USA). We registered the laboratory technique, the unit of measure of AAT, the type of sample (plasma or serum), if there was an identifiable standard for the quantification, the name of the laboratory kit used, and the name of the analyzer when available.

With this information, we performed a descriptive analysis using IBM SPSS Statistics (IBM Corporation, Armonk, NY, USA) version 26. Since all variables were qualitative, we performed a descriptive analysis of the absolute and relative frequencies of each category from the included variables.

## 3. Results

**Figure 1:**
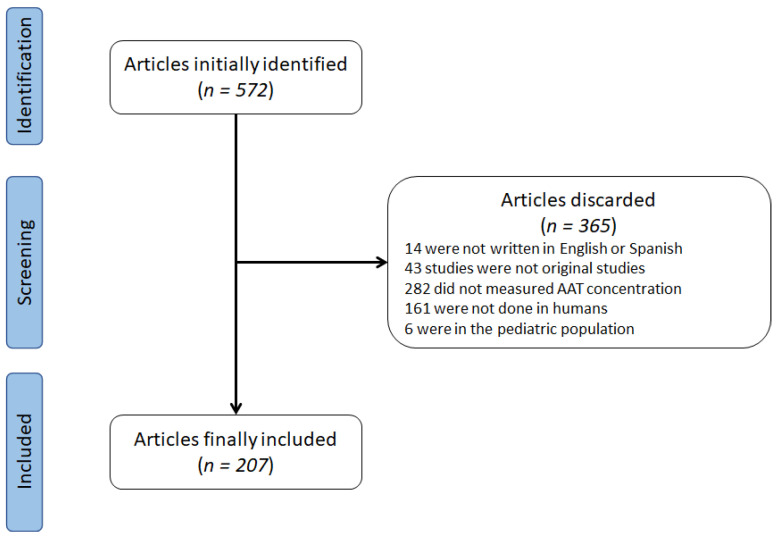
Flowchart of identification of studies.

The systematic search reported 572 articles fulfilling the pre-specified search (Figure 1). After the evaluation of the inclusion/exclusion criteria, 365 articles were excluded. The reasons for excluding these were: 14 were not written in English or Spanish, 43 studies were not original studies, 282 did not measure AAT concentration, 161 were not conducted on humans, and 6 were on the pediatric population. The final number of studies was therefore 207. Of these, two articles were impossible to obtain, and therefore we included the information available in the abstract.

Laboratory techniques used are summarized in Table 1 The most frequently used techniques were nephelometry, followed by Enzyme-linked immunosorbent assay (ELISA) and turbidimetry. There were four articles that combined two techniques: one article using radial immunodiffusion and nephelometry, one article using radial immunodiffusion and turbidimetry, one using rocket immunoelectrophoresis and nephelometry, and one using both nephelometry and turbidimetry. Altogether, nephelometry and turbidimetry immunoassays were used in 118 (57.0%) articles.

The units of measurements in the results were also highly variable as reflected in Table 2. The majority of the articles showed their results expressed as mg/dL, followed by g/L and mg/mL. Altogether, 182 (87.9%) cases expressed their results in gr or units of gr, while 16 (7.7%) articles expressed them in units of mole. The sample analyzed was 165 (79.7%) serum, 31 (14.9%) plasma, 1 article (0.4%) using both serum and plasma, and 10 (4.8%) using dried blood spot. There were no missing values in this variable.

The reference curve, the used commercial kit, or the analyzer was hardly available. Only 6 (2.9%) articles referred the standard used, 90 (43.5%) articles indicated the commercial kit used, and 75 (36.2%) indicated the analyzer used. In 67 (32.4%) cases, there was no information on the commercial kit nor the analyzer, and 25 (12.1%) articles showed both aspects.

## 4. Discussion

The present analysis describes the distribution of the different laboratory techniques, units of measurement, and samples for blood AAT level determination in the context of AATD. The results of the present study denote that there is a considerable variability in the techniques used but also in the units of measurement and samples processed. Of note, the differences in the methodology used could be taken into consideration when interpreting AAT results. Disgracefully, not all articles indicate these technical aspects of AAT determination, which should be considered a call for action for editors and reviewers of these journals.

AAT is a circulating glycoprotein synthesized and secreted mainly by hepatocytes whose major function is to protect lung tissues from damage caused for proteolytic enzymes such as neutrophil elastase. While risk for liver damage depends on the capability of the AAT variant to polymerize, the major determinant of risk for respiratory symptoms is AAT circulating levels [11,12]. Accordingly, quantitative tests evaluating AAT blood concentration have become a first step in numerous diagnostic algorithms [13]. Consequently, important health organizations such as the World Health Organization, the American Thoracic Society, or the European Respiratory Society strongly advise determining plasmatic AAT concentration in COPD and other AATD-associated conditions [14,15,16]. Notably, a plethora of laboratory methods have been used to diagnose AATD, whose technical peculiarities would be necessary to know.

An important issue to take into account when interpreting AAT concentration measures is that all quantitative tests need a standard with known concentration of AAT to plot a standard curve from which the unknown sample concentration is extrapolated. Therefore, accuracy of the standard is critical to obtain reliable measures and to compare between laboratories [17]. Unfortunately, the number of articles indicating the standard used in the determinations is alarmingly low. Even if we consider those works that indicate the commercial kit used, assuming that this standard is described in the technical requirements of the kit, the number is still considerably low.

Both nephelometry and turbidimetry are the most frequently used tests by far. Nephelometry is the preferred method in diagnosis guidelines [15]. It is based on the formation of antibody–antigen insoluble complexes that precipitate and disperse radiation when an intense beam of light passes through the sample. A nephelometer is needed to measure scattered light that is proportional to antigen quantity. A nephelometer comprises a light source that emit in the visible region a sample compartment, a photodetector placed at 90 degrees relative to the incoming light (I0), and a readout device. Currently, there are available instruments that allow automatization to provide laboratories with fast and accurate acquisition of nephelometric measures. Serum or plasma samples can be used, but it is really important to avoid hemolysis, lipemia, or frosting–defrosting cycles as turbidity is affected by these processes and could affect the results [18]. In the case of samples collected as dried blood spots, it is important to calculate the theoretical dilution of blood. To be in the working assay range, serum and plasma normally is diluted around 1:20, while blood eluted from dried blood spots can be diluted up to 1:64 [17]. Samples and serial dilutions of the standard are mixed with a polyclonal anti-human AAT antibody solution, and AAT concentration is measured by a nephelometer. Then, a standard curve is plotted to extrapolate sample concentration values taking into account the dilution made [11].

Turbidimetry follows the same physical principle as nephelometry, but in this case, what is measured is the decrease of the transmitted light intensity (absorbance) that is caused by the scattering provoked by antibody–antigen complexes. Thus, the amount of light transmitted without scattering is inversely proportional to AAT quantity present in the sample [19]. An improvement of this technique, the latex-enhanced turbidimetry assay, is less commonly used to diagnose AATD, but it is robust and comparable with nephelometry [15]. Serum or plasma AAT concentration can be measured, but for dried blood spot samples, nephelometry is recommended because it is preferred for low concentration determinations instead of turbidimetry [20]. Samples are processed such as for nephelometric assay and then absorbance is measured with a spectrophotometer [19].

In radial immunodiffusion, samples (serum or plasma) and standard are placed in a circular well punched into a gel matrix containing a specific antiserum to AAT and it is incubated for 24–48 h. While the sample diffuses through the gel, antibodies bind to AAT molecules and form a precipitated ring of antibody–antigen complexes that continue to grow until the equilibrium is reached. Precipitated ring diameter is dependent on AAT serum concentration. The diameter is measured manually and compared to the standard to calculate AAT concentration of the sample [21]. The main drawback of radial immunodiffusion is that commercially available standards tend to overestimate AAT concentration as much as 35–40%. This assay is capable to identify all PiZZ and PiSS homozygote individuals, but near of 15% of PiZ heterozygotes and up to 80% of PiMS present concentration values within the normal range, and 5% of normal individuals fall into the deficient range [22]. In addition, it is time- and temperature-dependent and requires skilled personnel to perform the assay [22]. Finally, nephelometry is a simpler method that has been shown to be more sensitive and more precise for quantitating serum proteins than radial immunodiffusion assay [23]. Therefore, although there are still some commercially available kits for AAT radial immunodiffusion assay, this diagnostic assay has been left behind by more reliable ones and it is no longer used for AATD diagnosis.

Rocket immunoelectrophoresis uses an agarose gel containing antibodies anti-AAT to precipitate plasma AAT and determine its concentration. Plasma aliquots and standards are loaded into the agarose gel and an electric field is applied. While proteins migrate through the gel, they bind with antibodies and precipitate, forming a precipitation zone that looks like ascending rockets. Gels are dried and stained with Comassie blue in order to measure distance between application well and head of the precipitate. The more AAT concentration in the sample, the more height of the precipitation peak in the gel. Standard curve is calculated and concentration of AAT in plasma can be extrapolated [24]. Rocket immunoelectrophoresis is fast, reproducible, simple, and flexible. The antigen needed is mildly less than with radial immunodiffusion. However, the accuracy of the technique relies on the quality of the standard used. Seronorm^TM^ is one of the standards used for determining AAT concentration in plasma samples [25,26].

XMAP technology was developed by Luminex Corporation to perform simultaneous quantitation of multiple analytes in one sample. Briefly, it is based on beads dyed with distinct fluorophores and associated to an antibody that is specific for one of the analytes of interest. Samples are combined with these dyed antibodies and then the assay solution is analyzed by the fluorometric array reader that detects fluorescence signal from each bead independently. This method allows for the identification of each analyte present in the sample and quantifies it by comparing it to a standard curve [27]. xMAP technology has been applied to determine AAT concentration in different samples such as serum, plasma, urine, or sputum. Aside from AATD patients [28], it has also been used to measure AAT concentration, along with other molecules, as a biomarker of disease severity in ulcerative colitis [29], long-term recovery of chronic disorder of consciousness [30], bladder cancer diagnosis signature [31], or inflammatory profile in chronic obstructive pulmonary disease [32].

Western blot is a general tool to specifically detect any protein according to their molecular weight and using specific antibodies. First, total proteins are separated by electrophoresis in a sodium dodecylsulfate polyacrylamide gel. Then, they are transferred to a polyvinylidene difluoride membrane and incubated with the specific primary antibody. Finally, membranes are incubated with a horseradish peroxidase-conjugated secondary antibody and revealed to detect protein bands by chemiluminescence. Normalizing band intensity against a reference, relative expression of the protein of interest can be calculated. AAT has been detected by Western blotting in serum samples from primary Sjögren’s syndrome patients using primary mouse anti-AAT monoclonal antibody (sc-69752, Santa Cruz Biotechnology, Dallas, Texas, USA). In this syndrome, AAT is 2.5 times less expressed than in healthy individuals [33].

Time-resolved immunofluorimetric assay has been used to detect trypsin-2–AAT complexes in serum samples from pancreatitis patients [34]. Microplate wells are coated with a monoclonal antibody anti-trypsin-2. Serum samples are added to these wells, allowing trypsin-2-AAT complex to bind to the antibodies. After washing, anti-AAT antibody labeled with a europium chelate is added. Then, an enhancement solution is added, and fluorescence emission of europium is measured [35]. The main advantages of time-resolved immunofluorimetric assay are the high fluorescence intensity achieved and the long fluorescence lifetime, which minimizes the background noise because excitation and emission do not occur simultaneously. However, a standard is needed to extrapolate protein concentration in the sample. The reference range for trypsin-2–AAT complexes in serum has been established as 2.3–12 μg/L [34].

ELISA is a highly extended technique to detect antigens in diverse samples. Its molecular base is similar to time-resolved immunofluorimetric assay. Antigens are immobilized to a plate surface, directly or using antibody coated plates, and then it is detected with labeled antibodies with a fluorophore or an enzyme. The main difference is the type of label used, because in the case of ELISA assay, detection reactions—fluorescence, colorimetric, or chemiluminescence—are fast, and readouts must be obtained in a short period of time. ELISA assay is highly sensitive and specific, is easy to perform, can use different biological samples, and is commercially available in high-throughput formats that allow for the analysis of multiple samples at a time. If a standard is added, normally included in the commercial kit, ELISA is a quantitative assay and antigen concentration can be determined. There are different commercial suppliers that offer ELISA kits to test AAT generally; to determine AAT concentration by ELISA, serum [36] or plasma [37] are used.

Diagnostic methods based on AAT immunoprecipitation using specific antibodies (immunoturbidimetry, immunonephelometry, radial immunodiffusion) or functional methods that measure the ability of the patient’s serum to inhibit neutrophils elastase (elastase inhibitory capacity tests) are commercially available and widely used in clinical settings [38]. However, these diagnostic methods face an additional problem. AAT is an acute phase reactant, and therefore plasma levels are modified in response to several pathophysiological conditions. On the one hand, plasma AAT levels are increased in individuals with active inflammatory/infectious processes [39], during pregnancy [40], and in women taking contraceptives [41]. On the other hand, AAT levels decrease in patients with liver disease and nephrotic syndrome [38]. In these cases, plasma AAT levels should not be determined during the inflammatory/infectious process. In any case, the interpretation of the results should consider C-reactive protein levels, especially in patients with inflammatory processes associated with COPD and liver disease. This problem can be solved by using additional diagnostic tests such as phenotyping and genotyping.

Plasma or serum are the traditional samples used to measure plasmatic AAT concentration. Additionally, dried blood spot has recently become a widespread method to collect biological samples for AAT determination. In this regard, dried blood spots have proven to be as reliable for quantifying AAT levels as serum or plasma [18,42]. The debate between serum and plasma continues to be an old and unsolved discussion. Although serum is still considered the gold standard for some studies, some authors point out some of its drawbacks such as processing time and the possible modification of some analytes due to the coagulation process [43]. Thus far, no work has evaluated whether there are notable differences between serum or plasma in the determination of AAT, and therefore they are considered equivalent. However, the question remains unexplored.

## 5. Conclusions

In conclusion, the present work analyzed the main techniques that have been described in the determination of AAT in blood in the scientific literature. Our results indicate that the technical aspects of these determinations are not always reported in the literature. However, these determinations may be influenced by the laboratory technique used. Specifically, it is necessary to indicate the devices used in the determination of AAT as well as the reference standards used. In the future, editors and reviewers should be attentive to these technical requirements and ensure that they are included in the works in which AAT is determined in order to ensure a correct interpretation of the study findings.

## Figures and Tables

**Table 1:** Laboratory techniques used in the revised articles.

**Laboratory Technique**	**Number of Studies** **(*n* = 207)**
Nephelometry	91 (43.9)
ELISA	41 (19.8)
Turbidimetry	28 (13.5)
Radial immunodiffusion	17 (8.2)
Immunofluorometry	4 (1.9)
Rocket immunoelectrophoresis	3 (1.4)
Luminex	2 (1.0)
Western blot	1 (0.5)
Not available	24 (11.6)

Results expressed as absolute and relative frequencies in parentheses. The sum of articles exceeds the number of articles included in the analysis because there were 4 articles that use two different techniques in the same work.

**Table 2:** Summary of the units of measurement of AAT levels.

Units of Measurement	Number of Studies (*n* = 207)
mg/dL	79 (38.2)
g/L	57 (27.5)
mg/mL	25 (12.1)
µmol/L (µM)	13 (6.2)
µg/L	5 (2.4)
ng/mL	4 (1.9)
mg/L	4 (1.9)
μg/mL	3 (1.4)
nM	3 (1.4)
µg/mL	3 (1.4)
mg%	1 (0.5)
g/dL	1 (0.5)
Not available	9 (4.3)

Results expressed as absolute and relative frequencies in parentheses.

## Data Availability

Data is contained within the article.
